# The E3 Ubiquitin Ligase HOIP inhibits Cancer Cell Apoptosis via modulating PTEN stability

**DOI:** 10.7150/jca.61996

**Published:** 2021-09-09

**Authors:** Zhiguo Niu, Xin Li, Shuxiao Dong, Jianhui Gao, Qingsong Huang, Huijie Yang, Hui Qian, Shu Zhuo, Ting Zhuang, Jian Zhu, Yinlu Ding, Wenrong Xu

**Affiliations:** 1 Jiangsu Key Laboratory of Medical Science and Laboratory Medicine, School of Medicine, Jiangsu University, Zhenjiang, Jiangsu, 212000, China.; 2 Henan Key Laboratory of Immunology and Targeted Drugs, School of Laboratory Medicine, Xinxiang Medical University, 453000, China.; 3 Department of Gastroenterology surgery, Shandong Provincial Third Hospital, Jinan, 250000, China.; 4 Signet Therapeutics Inc, Shenzhen, China. Research Institute of Tsinghua University in Shenzhen, Shenzhen, 518000, China.; 5 Department of general surgery, the Second Hospital, Cheeloo College of Medicine, Shandong University, Jinan, 250033, China.

**Keywords:** PTEN, HOIP, Apoptosis, Ubiquitin

## Abstract

Chemotherapy is widely used in a variety of solid tumors, such as lung cancer, gastric cancer and breast cancer. The genotoxic drugs, such as cisplatin, suppress cancer progression either by inhibition cell proliferation or facilitating apoptosis. However, the chemotherapy resistance remains an urgent challenge in cancer therapy, especially in advanced stages. Several studies showed that the activation of pro-survival pathways, such as PI3K-AKT, participated in mediating chemotherapy resistance. The insights into the molecular mechanisms for underlying chemotherapy resistance are of great importance to improve cancer patient survival in advanced stages. The HOIP protein belongs to the RING family E3 ubiquitin ligases and modulates several atypical ubiquitination processes in cellular signaling. Previous studies showed that HOIP might be an important effector in modulating cancer cell death under genotoxic drugs. Here, we report that HOIP associates with PTEN and facilitates PTEN degradation in cancer cells. Depletion of HOIP causes cell cycle arrest and apoptosis, which effects could be rescued by PTEN silencing. Besides, the survival data from public available database show that HOIP expression correlates with poor survival in several types of chemotherapy-treated cancer patients. In conclusion, our study establishes a novel mechanism by which HOIP modulates PTEN stability and facilitates chemotherapy resistance in malignancies.

## Introduction

Chemotherapy has been the major cancer treatment in advanced tumor stages 1. Most of the genotoxic drugs, such as cisplatin, are reactive, interact with DNA and induce DNA damage 2. Besides, they also induce tumor cell anti-proliferation and cancer cell apoptosis 3. Despite the progression of chemotherapy during the past 40 years, the improvement of long-term survival is still limited 4, 5. The side effects, including myelosuppression and nephrotoxicity, are still the restriction factors for the efficiency of chemotherapy 6. Based on the facts, it is a common aim for the oncological doctors to treat patients with improved efficiency, reduced side effects and effective control of acquired resistance.

There is a growing concern about the chemotherapy resistance, which compromises the therapy efficiency and severely hampers the long-term survival for cancer patients. Several intrinsic factors, including gene mutations, translocations, epigenetic changes, are proved to contribute drug resistance 7. Decreased drug uptake and increased efflux by the cell membrane transporters are also important mechanisms for compromised chemotherapy efficacy 8. Besides, the activation of pro-survival signaling and suppression of apoptotic pathways, including NFKB signaling and PI3K-AKT pathways, are proved to be important mediators for the development of chemotherapy resistance 9, 10. Although extensive efforts are put in investigating the potential mechanisms, still little is known about the determinants and pathways in mediating resistance. Hence, the chemotherapy resistance mechanisms and the development of corresponding overcome strategies are still subject to intensive studies.

Several studies have identified the PI3K-AKT pathway is hyper-activated in human cancers and facilitates chemotherapy resistance, which effect is counteracted by PTEN 11. The PTEN (Phosphatase and tensin homologue) is characterized as an important tumor suppressor, which inhibits growth factor signaling via PI3 kinase. The PTEN protein dephosphorylates phosphatidylinositol 3,4,5-trisphosphate (PIP3) and counter-balances the phosphorylation effect caused by AKT 12. The inactivation or mutation of PTEN has been connected to several human cancers, including inherited cancer (Hamartoma Tumor Syndromes) 13. Besides the genetic and epigenetic inactivation of PTEN, several studies also implicate the protein level of PTEN is always decreased through post-translational modifications, such as phosphorylation and ubiquitination 14, 15. Several ubiquitin ligases are shown to potentiate cancer progression via promoting PTEN ubiquitination and degradation, such as XIAP and RNF126 16, 17.

HOIP (HOIL-1-interacting Protein) belongs to the RING family protein family, which was firstly recognized as one component of the linear ubiquitin assembly complex (LUBAC) 18. Our previous studies showed that HOIP could facilitate estrogen signaling and suppress P53 pathway for breast cancer progression 19, 20. Interestingly, our further work reveals that HOIP modulates cisplatin-induced cell death regardless of P53 status, which means that HOIP could regulate chemotherapy sensitivity independent of P53 pathway. Here, our data show that HOIP correlates with poor chemotherapy outcome in several cancers. Molecular studies indicate that HOIP could associate with PTEN and facilitate its poly-ubiquitination and degradation, which ultimately prohibits chemotherapy-related inhibition in human cancers.

## Results

### HOIP correlates with chemotherapy sensitivity in several types of human cancers

**Figure 1:**
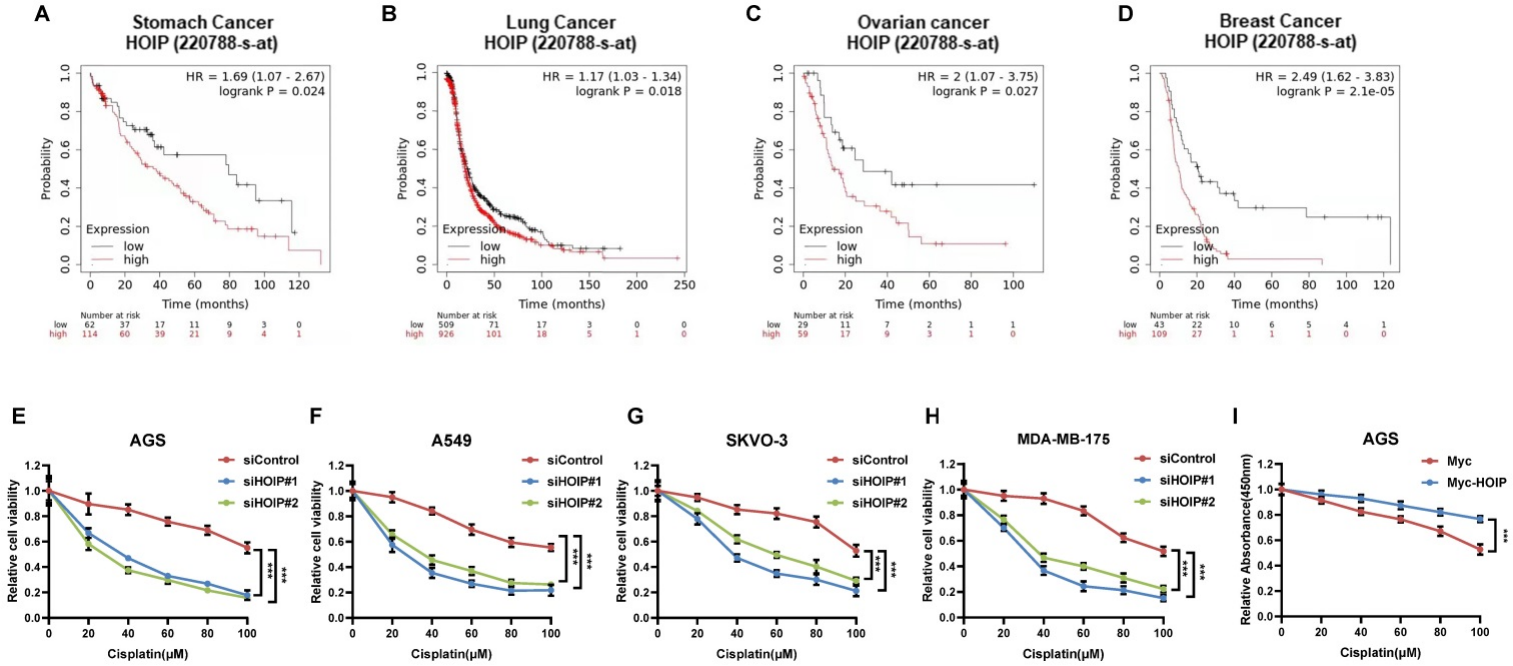
** HOIP correlates with chemotherapy sensitivity in several types of human cancers. A.** Kaplan-Meier graph of progression-free survival showed that HOIP relates to poor prognosis in gastric cancer patients treated with chemotherapy. **B.** Kaplan-Meier graph of progression-free survival showed that HOIP relates to poor prognosis in lung cancer patients treated with chemotherapy. **C.** Kaplan-Meier graph of progression-free survival showed that HOIP relates to poor prognosis in ovarian cancer patients treated with chemotherapy. **D.** Kaplan-Meier graph of progression-free survival showed that HOIP relates to poor prognosis in breast cancer patients treated with chemotherapy. **E.** HOIP depletion sensitized cisplatin-mediated cell inhibition in AGS cells. AGS cells were transfected with 50 nM siHOIP or siControl. After 24 hours, cells were treated with cisplatin for indicated concentration for 24 hours. The cell viability was determined via CCK8 assay. **F.** HOIP depletion sensitized cisplatin-mediated cell inhibition in A549 cells. A549 cells were transfected with 50 nM siHOIP or siControl. After 24 hours, cells were treated with cisplatin for indicated concentration for 24 hours. The cell viability was determined via CCK8 assay. **G.** HOIP depletion sensitized cisplatin-mediated cell inhibition in SKVO-3 cells. SKVO-3 cells were transfected with 50 nM siHOIP or siControl. After 24 hours, cells were treated with cisplatin for indicated concentration for 24 hours. The cell viability was determined via CCK8 assay. **H.** HOIP depletion sensitized cisplatin-mediated cell inhibition in MDAMB175 cells. MDAMB175 cells were transfected with 50 nM siHOIP or siControl. After 24 hours, cells were treated with cisplatin for indicated concentration for 24 hours. The cell viability was determined via CCK8 assay. **I.** HOIP over-expression conferred resistance in cisplatin-induced cell inhibition in AGS cells. AGS cells were transfected with 2 ug HOIP plasmid or empty vector. After 24 hours, cells were treated with cisplatin for indicated concentration for 24 hours. The cell viability was determined via CCK8 assay.

Since previous paper showed the correlation between HOIP and cisplatin resistance, we further analyze the HOIP effect in relation to the prognosis of chemotherapy patients 21. From the public available database, we observe that HOIP expression level correlates with poor survival in gastric cancer patients, who endured chemotherapy (Fig. 1A). Such conclusion is also confirmed in ovarian cancer, non-small cell lung cancer and breast cancer (Fig. 1B-D). Besides, we tested the correlation between HOIP expression and cisplatin sensitivity in a series of cancer cell lines, while HOIP was depletion by two independent siRNAs. The data showed that HOIP depletion could significantly sensitize cisplatin-induced cell death in AGS, A549, SKVO-3 and MDAMB175 cells (Fig. 1F-H). Besides, HOIP over-expression could further confer cisplatin-induced cell inhibition in AGS cells (Fig. 1I).

### HOIP deletion causes cell cycle arrest and inhibits cell growth in cancer

**Figure 2:**
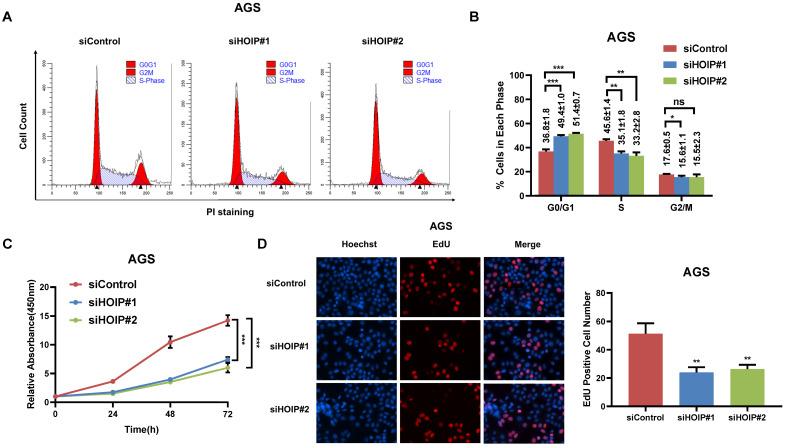
** HOIP deletion causes cell cycle arrest and inhibits cell growth in gastric cancer. A and B.** The cell cycle analysis of HOIP knockdown effect in AGS cells. AGS cells were transfected with 50nM HOIP siRNA or 50nM control siRNA. There were two independent siRNA be used. After 24 hours, cells were harvested, fixed via 70% ethanol and stained with propidium iodide. The cells were subject to FACS analysis. Experiments were done in triplicates. *P<0.05; ** P<0.01; ***P<0.001 for cell proportion comparison. The representative histograms and cell cycle phases were shown in Figure 2A and 2B respectively. **C.** HOIP depletion inhibited the cell proliferation in AGS cells. AGS cells were transfected with 50nM HOIP siRNA or 50nM control siRNA. There were two independent siRNA be used. After 24 hours, the CCK-8 assay was used to determine the cellar metabolic activity at indicated time points after transfection. Experiments were done in triplicates. *P<0.05; ** P<0.01; ***P<0.001 for cell growth comparison. **D.** HOIP depletion inhibited the number of EdU positive AGS cells. AGS cells were transfected with siControl or siHOIP. After 24 hours, EdU was added into the medium for 2 hours incubation. The absolute cell number was counted to indicate cell proliferation activity.

**Figure 3:**
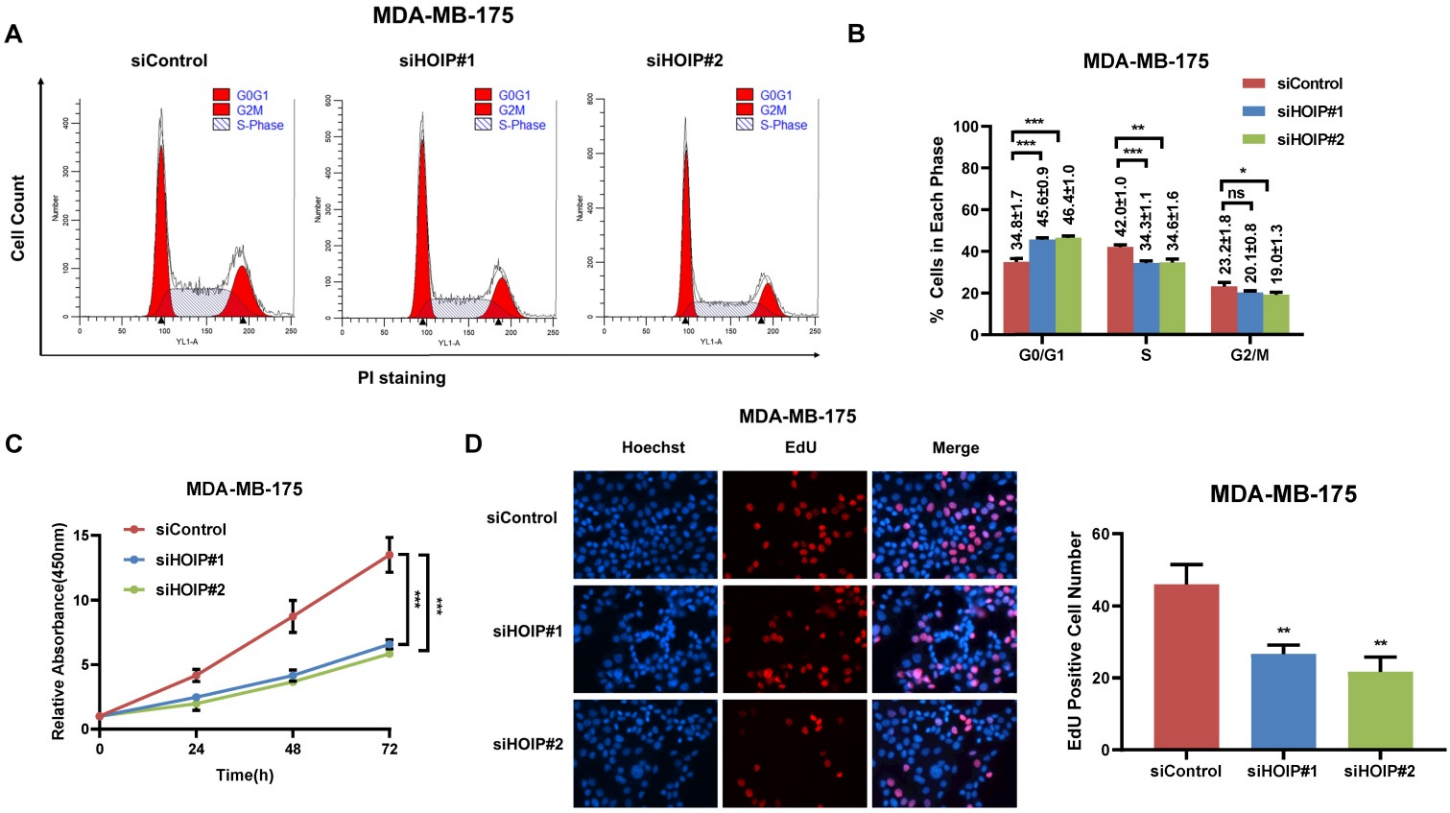
** HOIP deletion causes cell cycle arrest and inhibits cell growth in MDAMB175 cells. A and B.** The cell cycle analysis of HOIP knockdown effect in MDAMB175 cells. MDAMB175 cells were transfected with 50 nM HOIP siRNA or 50nM control siRNA. There were two independent siRNA be used. After 24 hours, cells were harvested, fixed via 70% ethanol and stained with propidium iodide. The cells were subject to FACS analysis. Experiments were done in triplicates. *P<0.05; ** P<0.01; ***P<0.001 for cell proportion comparison. The representative histograms and cell cycle phases were shown in Figure 3A and 3B respectively. **C.** HOIP depletion inhibited the cell proliferation in MDAMB175 cells. MDAMB175 cells were transfected with 50 nM HOIP siRNA or 50 nM control siRNA. There were two independent siRNA be used. After 24 hours, the CCK-8 assay was used to determine the cellar metabolic activity at indicated time points after transfection. Experiments were done in triplicates. *P<0.05; ** P<0.01; ***P<0.001 for cell growth comparison. **D.** HOIP depletion inhibited the number of EdU positive MDAMB175 cells. MDAMB175 cells were transfected with siControl or siHOIP. After 24 hours, EdU was added into the medium for 2 hours incubation. The absolute cell number was counted to indicate cell proliferation activity.

We utilize AGS cell as the model for the cell proliferation assays. The cell cycle analysis via flowcytometry shows that HOIP depletion significantly decreases the proportion of cells in S phase and increases the proportion of cells in G1 phase (Fig. 2A & B). The CCK8 assay implicates that HOIP depletion significantly inhibits cell growth in AGS cells (Fig. 2C). Besides, the EdU staining assay also indicates HOIP depletion significantly decreased the cell numbers of EdU incorporation in AGS cells (Fig. 2D). Besides, we further examined the HOIP depletion effect in MDAMB175 cells. The cell cycle analysis via flowcytometry shows that HOIP depletion significantly decreases the proportion of cells in S phase and increases the proportion of cells in G1 phase in MDAMB175 cells (Fig. 3A & B). The CCK8 assay implicates that in MDAMB175 cells, HOIP depletion significantly inhibits cell growth (Fig. 3C). Besides, the EdU staining assay also indicates HOIP depletion significantly decreased the cell numbers of EdU incorporation in MDAMB175 cells (Fig. 3D).

### HOIP deletion inhibits migration and causes cell apoptosis in gastric cancer

**Figure 4:**
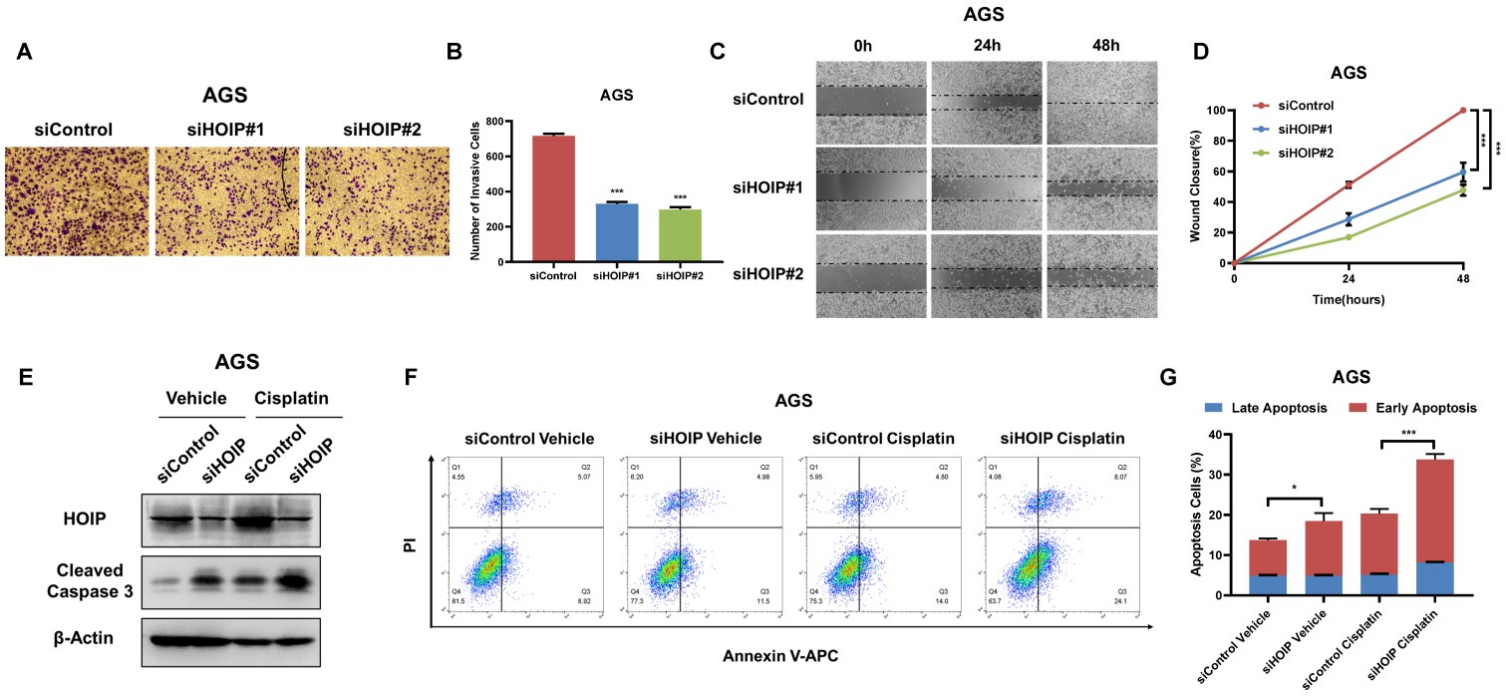
** HOIP deletion inhibits cell invasion and causes cell apoptosis in gastric cancer. A and B.** HOIP promotes cell invasion in AGS cells. AGS cells were transfected with indicated 50 nM HOIP siRNA (mix of #1 and #2) or 50 nM control siRNA. Trans-well was used to check the migration capacity. The cell number was counted and Data are presented as ±SD. **P<0.01, ***P<0.001 (student's t-test). **C and D.** Wound-healing assay of AGS cells were transfected with indicated 50 nM HOIP siRNA (mix of #1 and #2) or 50 nM control siRNA. Quantification of wound closure at the indicated time points. Data are presented as ±SD. **P<0.01, ***P<0.001 (student's t-test). **E.** HOIP depletion increased cleaved caspase-3 protein levels in AGS cells. AGS cells were transfected with siControl or siHOIP for 24 hours. Then cells were harvested for western blot analysis. HOIP and cleaved caspase-3 protein levels were determined by Western blot. Actin was used as internal control. **F and G.** HOIP depletion promoted apoptosis in AGS cells. AGS cells were transfected with siControl or siHOIP. After 24 hours, cells were stained with PI and Annexin V. Then cells were subject to FACS analysis for the proportion of apoptotic cells. Each group was done in triplicates. *P<0.05; ** P<0.01; ***P<0.001 for comparison.

Besides, HOIP silencing also inhibit cell invasion and migration capacity (Fig. 4A-D). We further utilize AGS cell as the model for the cell death assays. The immuno-blotting showed that HOIP depletion could increase cleaved caspase-3 level in AGS cells (Fig. 4E). We further confirm the conclusion in the Annexin V/PI double staining coupled with FACS analysis. The data shows that HOIP depletion increases the proportion of apoptotic cells in AGS cell line (Fig. 4F & G).

### HOIP modulates cell proliferation and apoptosis via PTEN in AGS cells

**Figure 5:**
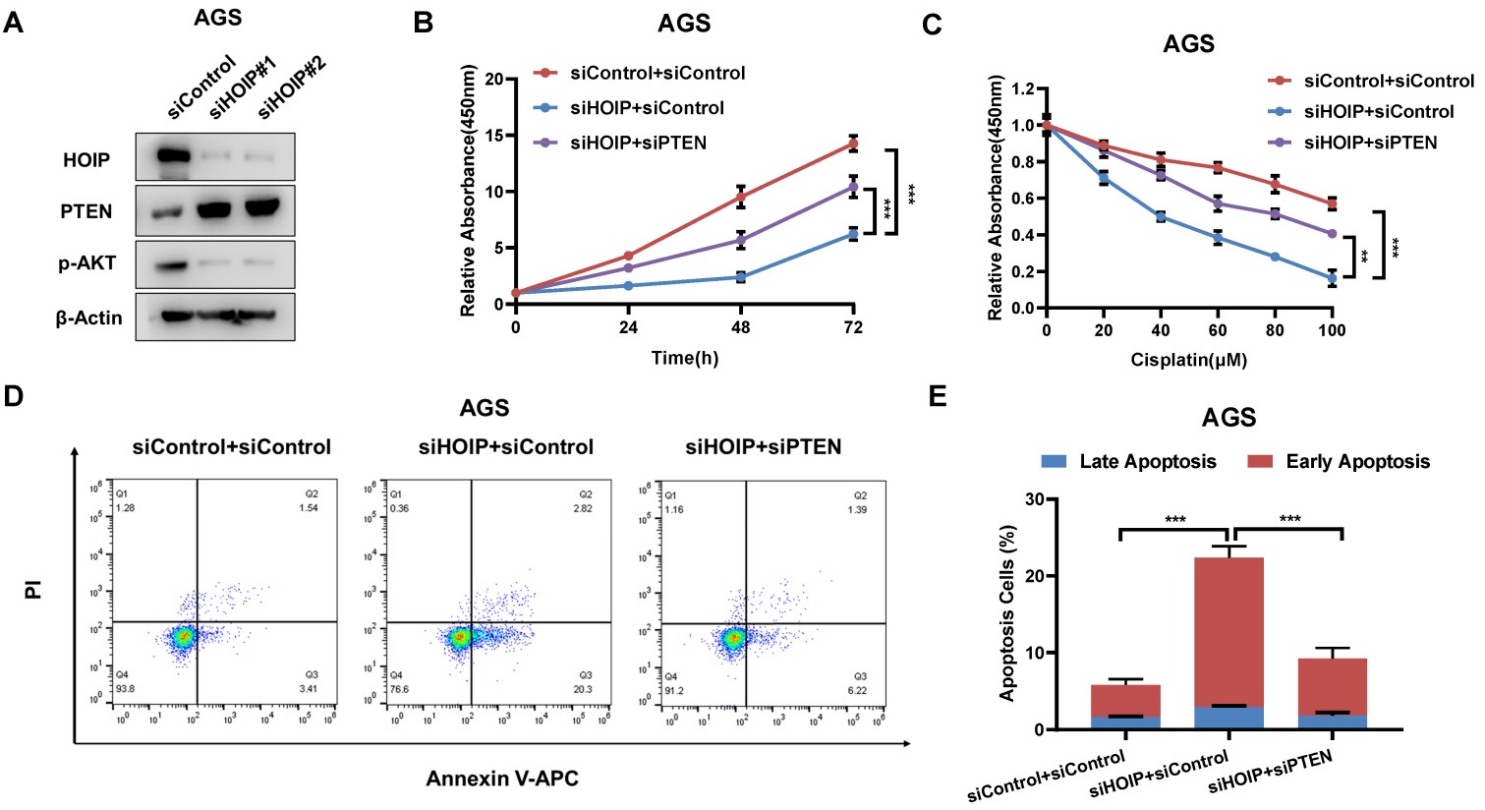
** HOIP modulates cell proliferation and apoptosis via PTEN in AGS cells. A.** HOIP depletion increase PTEN protein level, but decreases phosphor-AKT (Ser-473) in AGS cells. AGS cells were transfected with siControl or siHOIP. After 48 hours, cells were harvested for western blot analysis. HOIP, PTEN and phosphor-AKT (Ser-473) protein levels were determined by Western blot. Actin was used as internal control. **B.** Cell growth inhibition by HOIP silencing could be partially rescued by PTEN depletion in AGS cells. AGS cells were transfected with 50nM HOIP siRNA, 50nM control siRNA or 50nM siHOIP+siPTEN. After 24 hours, the CCK-8 assay was used to determine the cellar metabolic activity at indicated time points after transfection. Experiments were done in triplicates. *P<0.05; ** P<0.01; ***P<0.001 for cell growth comparison. **C.** HOIP depletion sensitized cisplatin-induced inhibition in AGS cells, which effect could be partially rescued by PTEN depletion. AGS cells were transfected with 50nM HOIP siRNA, 50nM control siRNA or 50nM siHOIP+siPTEN. The cells were treated with cisplatin for indicated concentration for 24 hours. The cell viability was determined via CCK8 assay. **D and E.** HOIP depletion promoted apoptosis, which effects could be partially rescued by PTEN depletion in AGS cells. AGS cells were transfected with 50nM HOIP siRNA, 50 nM control siRNA or 50nM siHOIP+siPTEN. After 24 hours, cells were stained with PI and Annexin V. Then cells were subject to FACS analysis for the proportion of apoptotic cells. Each group was done in triplicates. *P<0.05; ** P<0.01; ***P<0.001 for comparison.

In order to investigate the logic link between the cancer phenotype and PTEN in HOIP function, we carry out several rescue experiments. The immuno-blotting assay shows that HOIP depletion increases PTEN protein level, but decreases the phosphorylation of AKT (S473) (Fig. 5A). Figure 4B shows that HOIP depletion could inhibit AGS cell proliferation, which effect could be at least partially rescued by PTEN knocking-down (Fig. 5B). Besides, the HOIP depletion could sensitize cisplatin-caused cell inhibition, which effect could be partially rescued by PTEN silencing (Fig. 5C). The annexin V/PI staining coupled with FACS analysis shows that PTEN silence could at least partially rescue cell apoptosis caused by HOIP depletion in AGS cells (Fig. 5D & 5E).

### HOIP associates with PTEN and modulates PTEN stability

**Figure 6:**
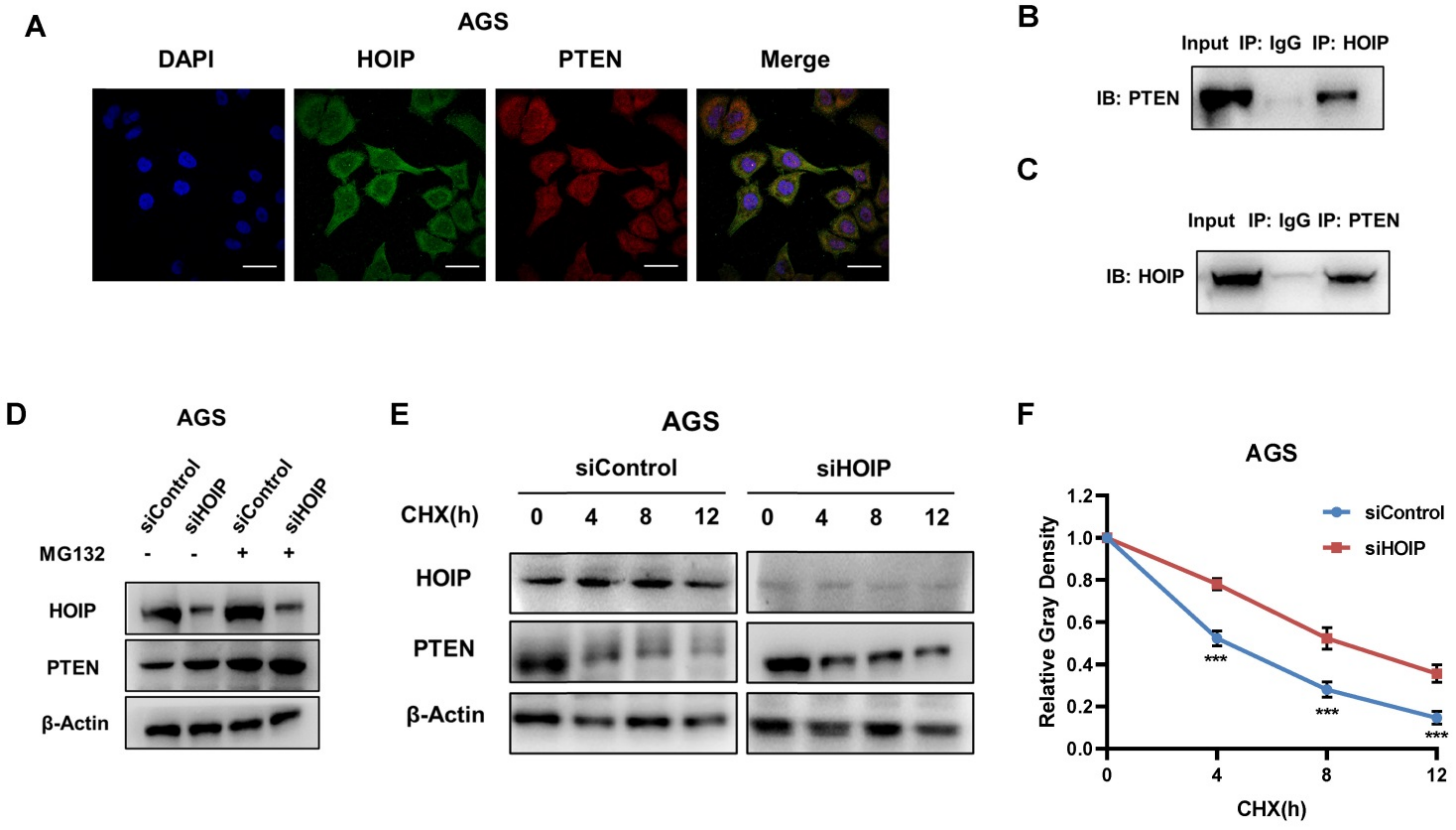
** HOIP associates with PTEN and modulates PTEN stability. A.** Intracellular localization analysis of HOIP and PTEN by immunofluorescence assay. AGS cells were cultured in normal medium before fixation. Intracellular localization of PTEN (red) and HOIP (green) were shown. Nuclei (blue) were stained with 40,6-diamidino-2-phenylindole (DAPI). **B and C.** Co-IP assay reveals association between endogenous HOIP and PTEN in AGS cells. AGS cells were harvested with RIPA lysis buffer. CO-IP was performed using antibody as indicated. **D.** In the presence of the proteasome inhibitor MG132, HOIP could not further increase PTEN protein levels. AGS cells were transfected with 50 μM siControl or siHOIP. After 24 h, cells were treated with 10 µM MG132/vehicle for 6h. Cell lysates were prepared for Western blot analysis. The results are representative for three independent experiments. **E and F.** HOIP depletion increases PTEN half-life in AGS cells. AGS cells were transfected with 50nM siControl or siHOIP. After 24 h, cells were treated with 100 µM cycloheximide/vehicle for indicated times. Cell lysates were prepared for Western blot analysis. The results are representative for three independent experiments. The PTEN relative density was measured by Image J software.

We further investigate the regulatory mechanism between HOIP and PTEN. The immuno-staining shows that HOIP is mainly located in the cytosol, while PTEN is located both in cytosol and nucleus (Fig. 6A). The endogenous immuno-precipitation shows that HOIP could interact with PTEN in AGS cells (Fig. 6B & C). HOIP depletion could increase PTEN protein level, which effect could be diminished via the proteasome inhibitor MG132 (Fig. 6D). This might indicate that HOIP modulates PTEN via protein stability. Then we utilized cycloheximide, the protein synthesis inhibitor, to measure the protein stability. Figure 6E & F shows that HOIP depletion significantly increased endogenous PTEN half-life (Fig. 6E & F).

### HOIP promotes PTEN K48-linked poly-ubiquitination and degradation

**Figure 7:**
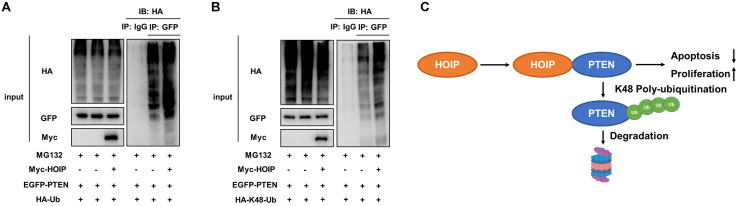
** HOIP promotes PTEN K48-linked poly-ubiquitination and degradation. A.** HOIP increases total poly-ubiquitination of PTEN. HEK293 cells were transfected with 2 µg EGFP-PTEN plasmid, 0.5 µg HA-Ub plasmid and 0.5 µg Myc-tag or Myc-HOIP plasmids. The cell extracts were immunoprecipitated with HA antibody. The total poly-ubiquitinated PTEN was detected via western blotting analysis. **B.** HOIP increases K48-linked poly-ubiquitination of PTEN. HEK293 cells were transfected with 2 µg EGFP-PTEN plasmid, 0.5 µg HA-K48 Ubi plasmid and 0.5 µg Myc-tag or Myc-HOIP plasmids. The cell extracts were immunoprecipitated with HA antibody. The total poly-ubiquitinated PTEN was detected via western blotting analysis. **C.** The hypothetical model for HOIP regulating PTEN in cancers: HOIP interacts with PTEN protein and directly promotes PTEN K48-linked poly-ubiquitination and degradation, which subsequently promotes cell cycle arrest and apoptosis.

As on kind of E3 ubiquitin ligase, previous studies show that HOIP could facilitate several ubiquitination manners, such as K48-linked ubiquitination, linear ubiquitination and mono-ubiquitination. We further investigate the effect of HOIP on PTEN ubiquitination. The ubiquitin-based immuno-precipitation assay shows that HOIP could facilitate the overall and K48-linked poly-ubiquitination level of PTEN (Fig. 7A & B).

## Discussion

In our study, we identify the RING finger protein HOIP could interact with PTEN protein and promote PTEN poly-ubiquitination and degradation in human cancers (Fig. 7C). In addition, HOIP depletion could induce cell cycle arrest and cisplatin-induced apoptosis, which effect could be partially rescued by PTEN silencing. These data implicate that targeting HOIP could be a promising strategy to reverse or alleviate chemotherapy resistance.

Chemotherapy is the most important and commonly used regimen for human cancers, especially for recurrent or metastatic cases. The potential mechanisms for chemotherapy resistance are complicated. For example, cancer cells could increase the efflux or decrease the influx of chemotherapy drug, which protect the damage to the cells. Besides, the activation of DNA repair process and autophagy are also critical when the cancer cells endure chemotherapy stress 22. Interestingly, several studies implicate that the activation of pro-survival and inactivation of apoptotic pathway also play critical roles in chemotherapy 23. For example, the normal function of P53 is necessary for cisplatin-induced cell death, while the mutation or deletion of P53 could lead the tolerance of DNA damage and impair the cellular apoptotic response caused by chemotherapy 24. Besides, the activation of AKT signaling and the loss of PTEN also prove to be important in mediating chemotherapy resistance 25.

PTEN is a ubiquitously expressed tumor suppressor, which counteracts the activation of AKT through its lipid phosphatase activity and inhibits MAPK signaling via its protein phosphatase activity 12, 26. PTEN abnormalities are widely observed in human cancers, such as loss of heterozygosity (LOH) or gene mutations 27, 28. However, recent studies also report that the post-translational modifications of PTEN also have functional impacts in cancer behaviors, such as cancer progression and drug resistance 29. For example, the HECT family E3 ubiquitin ligase SMURF1 could promote PTEN poly-ubiquitination and degradation, which subsequently facilitates cancer progression in glioblastoma 30. In our current study, we implicate the RING Family E3 ligase HOIP in mediating cancer growth and cisplatin resistance via facilitating PTEN degradation. Our study provides a novel regulation mechanism in modulating PTEN stability by HOIP and provides potential targets to re-sensitize chemotherapy resistance in human cancers.

HOIP gene was firstly cloned from MCF-7 cells in 2004 31. Further studies revealed that HOIP protein is highly expressed in muscles, testis and heart 32. The whole-body knockout of HOIP will lead to embryonic lethality by TNFR-mediated cell death 33. The most well studied function of HOIP is that it could form the linear ubiquitination assembly complex (LUBAC) with RBCK1 and SHARPIN, which facilitates the linear ubiquitination of IKKγ and the signaling transduction of NFKB 34. Our previous study showed that HOIP could promote ER alpha signaling and suppress P53 signaling in breast cancer cells 35. Although HOIP could inhibit wild type P53 function and facilitate cell survival, our current study shows that HOIP could still promote cell growth and inhibit apoptosis in P53 mutant background. Our molecular experiments show that HOIP could exert its pro-survival function via mediating PTEN degradation.

In conclusion, our study demonstrates the E3 ligase HOIP as a regulator of PTEN/AKT signaling in human cancer cells. HOIP suppresses PTEN protein level and promotes breast cancer cell growth and anti-apoptosis. As a newly discovered modulator of PTEN, HOIP could be a promising target to sensitize chemotherapy or reverse chemotherapy resistance in human cancers.

## Materials and Methods

### Cell culture

AGS, MDAMB175, HEK293, A549 and SKVO-3 cells are acquired form American Type Culture Collection (ATCC). A549 and AGS cells are maintained with RPMI-1640 (42401, Life Technologies) supplemented with 2 mM L-glutamine (25030, Life Technologies) and 10% FBS. MDAMB175, HEK293 and SKVO-3 cells are culture with Dulbecco's Modified Eagle's Medium that contains 4.5 g/L glucose and 4 mM L-glutamine (DMEM, 41965, Life Technologies) supplemented with 10% Fetal Bovine Serum (FBS, 10270, Life Technologies). All cell lines are characterized by cell line authentication. The cell line authentication via Short Tandem Repeat (STR) is performed via PowerPlex 21 system. The STR data of A549, AGS, MDAMB175, SKVO-3 and HEK293 cell lines are found consistent with STR data in ATCC.

### Western blot

Standard western-blot assays are used to analyze protein expression in cells. The following antibodies are used for assays: anti-GFP (AB290, Abcam, 1:1000), anti-HA (901514, Biolegend, 1:1000) anti-Myc (AB32, Abcam, 1:1000), anti-Actin (3700, Cell Signaling Technology, 1:1000), anti-HOIP (Ab46322, Abcam, 1:1000), anti-PTEN (9552, Cell Signaling Technology, 1:1000), anti-Phospho-AKT (9271, Cell Signaling Technology, 1:1000) and anti-Cleaved Caspase-3 (9661, Cell Signaling Technology, 1:1000). Protein signals are detected with an ECL kit (Millipore Co., Billerica, Massachusetts, USA).

### Plasmids and siRNA

The Myc-HOIP plasmid is acquired from our previous study 19. The PTEN plasmid is acquired from the Addgene platform. The HA-K48 and HA-K63 Ubi plasmids were used in previous study 36. The plasmids are transfected with Lipofectamin 2000 (1662298, Invitrogen). For siRNA transfection, the HOIP siRNA sequences are provided in the previous study 20. The PTEN siRNA sequences are shown: 5-GUU AGC AGA AAC AAA AGG AGA UAU CAA-3, 5-UUG AUA UCU CCU UUU GUU UCU GCU AAC-3. The siControl sequence is 5-UUC UCC GAA CGU GUC ACG UTT-3, 5-ACG UGA CAC GUU CG GAGA ATT-3.

### Quantification of cell viability

AGS, MDAMB175, A549 and SKVO-3 cells are transfected with siHOIP or siControl into 24-well plates. Twenty-Four hours after transfection, the cells number was countered and 4000 cells were seeded into 96-well plates. The relative cell viability was measured at indicated time points. Cell numbers are determined using the CCK8 cell proliferation reagent with the absorbance at 450 nm. For cisplatin-induced cell death assay, AGS, MDAMB175, A549 and SKVO-3 cells are transfected with siHOIP or siControl into 24-well plates. Twenty-Four hours after transfection, the cells number was countered and 20000 cells were seeded into 96-well plates. Different concentrations of cisplatin are added for 24 hours. Cell viability is determined using the CCK8 cell proliferation reagent with the absorbance at 450 nm.

### EdU staining assay

For ethynly-deoxyuridine (EdU) labeled DNA, cells were incubated with EdU for 2 hours. Later on, the cells were fixed in cell culture plates with 4% formalin. The EdU positive cells were counted with statistical analysis.

### Flow cytometry assay

For the cell cycle analysis, the AGS cells were transfected with 50 uM siHOIP or siControl. After 24 hours, cells were fixed via 70% ethanol and stained with propidium iodide. For the apoptosis assay, the AGS cells were transfected with 50 uM siHOIP or siControl. Twenty-four hours post-transfection, cells were stained with propidium iodide and annexin V. The BD LSR flow was used to measure the fluorescence intensity.

### Co-immunoprecipitation assay

Immunoprecipitation is performed as described in previous study 37. The AGS total cell lysis is pre-cleared with rabbit IgG for 2 h and subsequently immunoprecipitated with HOIP antibody (Ab46322, Abcam) over night, while rabbit IgG (Santa Cruz) is used as the negative control. The bounded protein is analyzed by Anti-PTEN (9552, Cell Signaling Technology). The bound proteins are analyzed by western blotting.

### Protein stability assays

About 10^5^ AGS cells are seeded into twenty-four well plates and transfected with 50 μM HOIP siRNA or siControl. After 48h, cells are treated with 100μM cycloheximide (C7698, Sigma) for indicated time points. Samples are subject to western blot for PTEN degradation.

### Poly-ubiquitination detection assay

To directly detect the enriched K48-ubiquitinated and total ubiquitination PTEN from the cell extracts, HEK293 cells are transfected with K48 Ubi or Ub plasmids together with EGFP-PTEN plasmid and Myc-HOIP or Myc-vector. After 24 h, the cells are treated with 20 μM MG132 for 7 hours, then total protein is extracted and pre-cleared with 30μl protein A (santa cruz, SC-2001) for 4 h. The supernatant is collected and immunoprecipitated by PTEN antibody. Western blot with HA antibody is performed to detect total poly-ubiquitinated PTEN or K48-linked poly-ubiquitinated PTEN.

### Statistical analysis

Statistical analysis is performed by GraphPad Prism 7 software or SPSS version 23.0 (SPSS, Inc., IL). Data are expressed as mean ± s.e.m. Differences between two independent groups were tested with Student's t-test. Kaplan-Meier analysis with log-rank test was applied for survival analysis. Differences are considered to be statistically significant when *P < 0.05; **P < 0.01; ***P < 0.001.

## Abbreviations

PTEN: Phosphatase and tensin homologue
HOIP: HOIL-1-interacting Protein
PIP3: Phosphatidylinositol 3,4,5-trisphosphate
LUBAC: the linear ubiquitin assembly complex
NFKB: Nuclear factor kappa B
ATCC: American Type Culture Collection
STR: Short Tandem Repeat
IP: Immuno-precipitation
